# Association of Frailty Index at 66 Years of Age with Health Care Costs and Utilization Over 10 Years in Korea: Retrospective Cohort Study

**DOI:** 10.2196/50026

**Published:** 2025-01-27

**Authors:** Jieun Jang, Anna Kim, Mingee Choi, Ellen P McCarthy, Brianne Olivieri-Mui, Chan Mi Park, Jae-Hyun Kim, Jaeyong Shin, Dae Hyun Kim

**Affiliations:** 1 Department of Preventive Medicine, College of Medicine, Dongguk University, Gyeongju, Republic of Korea; 2 School of Economics, Yonsei University, Seoul, Republic of Korea; 3 Department of Preventive Medicine, Yonsei University College of Medicine, 50 Yonsei-ro, Seodaemun-gu, Seoul, 03722, Republic of Korea, 82 2-2228-1881; 4 Frailty Research Center, Hinda and Arthur Marcus Institute for Aging Research, Hebrew SeniorLife, Harvard Medical School, Boston, MA, United States; 5 Division of Gerontology, Department of Medicine, Beth Israel Deaconess Medical Center, Harvard Medical School, Boston, MA, United States; 6 Department of Health Sciences, Bouvé College of Health Sciences, Northeastern University, Boston, MA, United States; 7 Department of Health Administration, College of Health Science, Dankook University, Cheonan, Republic of Korea; 8 Department of Policy Analysis and Management, College of Human Ecology, Cornell University, Ithaca, NY, United States

**Keywords:** frailty index, health care costs, health care utilization, elderly, Korea, frailty, aging, utilization, older adults, sociodemographic, cost, prevention

## Abstract

**Background:**

The long-term economic impact of frailty measured at the beginning of elderhood is unknown.

**Objective:**

The objective of our study was to examine the association between an individual’s frailty index at 66 years of age and their health care costs and utilization over 10 years.

**Methods:**

This retrospective cohort study included 215,887 Koreans who participated in the National Screening Program for Transitional Ages at 66 years of age between 2007‐2009. Frailty was categorized using a 39-item deficit accumulation frailty index: robust (<0.15), prefrail (0.15‐0.24), and frail (≥0.25). The primary outcome was total health care cost, while the secondary outcomes were inpatient and outpatient health care costs, inpatient days, and number of outpatient visits. Generalized estimating equations with a gamma distribution and identity link function were used to investigate the association between the frailty index and health care costs and utilization until December 31, 2019.

**Results:**

The study population included 53.3% (n=115,113) women, 32.9% (n=71,082) with prefrailty, and 9.7% (n=21,010) with frailty. The frailty level at 66 years of age was associated with higher cumulative total costs (robust to frail: $19,815 to $28.281; *P*<.001), inpatient costs (US $11,189 to US $16,627; *P*<.001), and outpatient costs (US $8,625 to US $11,654; *P*<.001) over the next 10 years. In the robust group, a one-year increase in age was associated with increased total health care costs (mean change per beneficiary per year: US $206.2; SE: $1.2; *P*<.001), inpatient costs (US $126.8; SE: $1.0; *P*<.001), and outpatient costs (US $74.4; SE: $0.4; *P*<.001). In the frail group, the increase in total health care costs was greater compared to the robust group (difference in mean cost per beneficiary per year: US $120.9; SE: $5.3; *P*<.001), inpatient costs (US $102.8; SE: $5.22; *P*<.001), and outpatient costs (US $15.6; SE: $1.5; *P*<.001). Similar results were observed for health care utilization (*P*<.001). Among the robust group, a one-year increase in age was associated with increased inpatient days (mean change per beneficiary per year: 0.9 d; *P*<.001) and outpatient visits (2.1 visits; *P*<.001). In the frail group, inpatient days increased annually compared to the robust group (difference in the mean inpatient days per beneficiary per year: 1.5 d; *P*<.001), while outpatient visits increased to a lesser extent (difference in the mean outpatient visits per beneficiary per year: −0.2 visits; *P*<.001).

**Conclusions:**

Our study demonstrates the potential utility of assessing frailty at 66 years of age in identifying older adults who are more likely to incur high health care costs and utilize health care services over the subsequent 10 years. The long-term high health care costs and utilization associated with frailty and prefrailty warrants public health strategies to prevent and manage frailty in aging populations.

## Introduction

Health care systems face the challenge of managing increasing health care costs in aging populations [1,2]. South Korea experiences one of the fastest rates of population aging in the world, with 40% of its population expected to be aged over 65 years by 2050 [3]. The country is already experiencing a deficit in health insurance budget [4]. Proactive identification of individuals who are likely to incur high health care costs and utilization is critical for developing strategies to control health care expenditures in the aging society.

Frailty is defined by a decline in physiological reserves across multiple organ systems, leading to increased susceptibility to poor health outcomes following stressors [5]. Previous studies have found that frailty was associated with increased health care costs and utilization [6-10]. Moreover, individuals with frailty accounted for 43.9% of preventable health care expenditures [11]. One of the established models for measuring frailty is the deficit accumulation frailty model, expressed as a frailty index (FI), which is calculated by dividing the number of deficits present by the total number of deficits considered [12,13]. The FI can range from 0 to 1, with higher scores indicating greater frailty [13]. It can be derived from an existing database that contains information on standardized health assessments.

Since 2007, all Koreans who turn 66 years old have been invited to participate in a comprehensive health evaluation as part of the National Screening Program for Transitional Ages at government-approved clinics, hospitals, and public health facilities [14]. This examination assesses lifestyle, medical history, functional status, and laboratory tests, providing data to quantify a deficit accumulation FI on a national level. We have recently shown that higher FI scores at age 66 were associated with faster development of age-related chronic diseases over the subsequent 10 years among nearly 1 million Koreans [15]. The availability of standardized health assessments through the National Screening Program for Transitional Ages provides a unique opportunity to identify the FI across a large cohort. However, it is uncertain whether the FI at age 66 can predict long-term health care costs and utilization.

We conducted a nationwide cohort study of Koreans who participated in the National Screening Program for Transitional Ages in 2007‐2009 to examine the association between the FI at age 66 and subsequent health care costs and utilization over 10 years. South Korea offers a unique context for research in frailty due to its nationwide single-payer health insurance system, which ensures both universal coverage, and provides detailed and standardized claims data for the entire population. This system facilitates long-term follow-up of health care costs and utilization, enabling population-level analyses that are rarely feasible in other health care settings. In this study, we hypothesized that a higher FI at age 66 would be associated with a greater increase in health care costs and utilization over 10 years.

## Methods

### Data Sources

We accessed the National Screening Program for Transitional Ages database (2007‐2009), which was linked to the Korean National Health Insurance database (2004‐2019), through the Korean National Health Insurance Corporation research program. The dataset included a 35% (n=435,572) random sample of adults who reached the age of 66 years between 2007-2009. The screening program database includes information on lifestyle indicators, medical history, functional status, and laboratory measurements. The Korean National Health Insurance database includes ICD-10 diagnostic codes, sociodemographic variables, health service claims, health care utilization, and long-term care insurance claims [16].

### Study Population

We included individuals with complete sociodemographic information who participated in the screening program (n=222,480) through the 435,572 enrollees of the Korean National Health Insurance aged 66 years between 2007‐2009. We excluded those with (1) duplicate records (n=1460), (2) insufficient data (<80% of the necessary items) for calculating frailty (n=5119), or (3) death within the month of examination or the month following the examination (n=14). Our final cohort included 215,887 enrollees (Figure S1 in Multimedia Appendix 1). This cohort included both community-dwelling older adults and long-term care residents.

### Measurement of Frailty and Other Characteristics

The detailed procedure for constructing the FI has been described previously [15]. Briefly, we followed the standard procedure established by Searle et al [17]. The variables were selected as health deficits if they met the following criteria: (1) associated with health status, (2) prevalence increased with age, (3) did not saturate too early, and (4) covered a range of organ systems. We calculated the FI (range: 0 to 1; higher scores indicated greater frailty) based on 39 health-deficit items in the following health domains: medical history (15 items), biometric or laboratory measures (8 items), physical health (2 items), psychological health (8 items), and disability (6 items) (the definition of each item is provided in Table S1 in Multimedia Appendix 1) [15]. Frailty categories were defined using the previously used cut off points [15,18,19]: robust (<0.15), prefrail (0.15 to <0.25), and frail (≥0.25). We also assessed the presence of chronic conditions using ICD-10 diagnosis codes (ie, 1 inpatient or 2 outpatient diagnoses) from the previous year. Additionally, the following characteristics were obtained from the screening examination: sex, annual income level (quantiles), insurance status (employee insurance, self-employed insurance, or medical aid for low income), residential area (capital, metropolitan, or rural areas), and examination year (2007, 2008, or 2009).

### Outcome Measurements and Follow Up

The primary outcome was total health care costs per beneficiary per year. These health care costs were the sum of reimbursements from the Korean National Health Insurance and beneficiaries’ cost-sharing for inpatient and outpatient care. Secondary outcomes were inpatient costs, outpatient costs, inpatient days, and the number of outpatient visits per beneficiary per year. Follow up began on the day after the screening examination and lasted until the following, depending on whichever occurred earlier: date of death, 10 years from the screening examination, or December 31, 2019. To account for variations in inflation, we adjusted the costs to 2007 Korean Won (KRW) using an annual conversion factor (converted into US dollars at an exchange rate of 1 USD=1200 KRW). The extreme cost values were truncated at the 1st and 99th percentiles (ie, replacing values smaller than the 1st percentile with the 1st percentile value, and values larger than the 99th percentile with the 99th percentile).

### Statistical Analysis

We used the *χ*^*2*^ test to compare baseline characteristics by frailty category. The cumulative annual costs (total health care costs, inpatient costs, and outpatient costs) were compared using one-way ANOVA. Additionally, we compared the annual rates of health care utilization (inpatient days and number of outpatient visits) over 10 years, according to the frailty category. To investigate the association between the FI at age 66 years and health care costs and utilization over 10 years, generalized estimating equations were applied with a gamma distribution and the identity link function [20,21]. This was done to model right-skewed and over-dispersed health care cost and utilization data, accounting for repeated annual measures of costs and utilization within each beneficiary. Subgroup analyses were conducted by sex and by health insurance type. We tested the homogeneity of the interaction term between time and frailty categories across sex and health insurance type [22,23]. All models were adjusted for sex, annual income, insurance status, residential area, and examination years.


$$\begin{array}{l} Y_{i\tau }=B_{0}+B_{1}*Prefrail_{i\tau }+B_{2}*frail_{i\tau }+B_{3}*Time_{i\tau }+B_{4}*Time_{i\tau }*Prefrail_{i\tau }+\\ B_{5}*Time_{i\tau }*Frail_{i\tau }+B_{6}*X_{i}+e_{i\tau } \end{array}$$

• *Y*_*iT*_: Dependent variable for an individual participant *i* observed for time *T*

• *T=t–c*; where time *T* is defined as year *t* minus the calendar year in which an individual participant *i* underwent a medical examination year *C^i^* (2007, 2008, or 2009)

• Time*_t_*: Time in years [0 (67 years), 1 (68 years), 2 (69 years),…, 9 (76 years)]

• Prefrail_*iT*_: Dummy variable (1 for prefrail group, 0 otherwise)

• X_*i*_: Independent variables

The analyses were performed using SAS Enterprise Guide (version 7.15; SAS Institute,) and STATA (version 15; Stata Corporation). A two-sided *P*-value <.05 was considered statistically significant.

### Ethical Considerations

This study was exempted from review by the Institutional Review Board of Ajou University Health System (AJIRB-MED-EXP-20‐127) as the data used in this study were deidentified and secondary analysis was performed using public data. No compensation was provided to the participants.

## Results

The study population included 123,795 (57.3%) women, 71,082 individuals with prefrailty (32.9%), and 21,010 individuals with frailty (9.7%) (Table 1). Individuals with greater frailty were more likely to be women (robust vs frail: 44.8% vs 68.6%) and medical aid recipients (robust vs frail: 1.8% vs 7.8%). Over the 10-years follow up, 20,189 (9.4%) individuals died. The survival rate to age 76 years was higher in the robust group than in the prefrail and frail groups (robust: 91.8%; pre-frail: 90.3%; frail: 85.2%) (Table S2 in Multimedia Appendix 1).

**Figure 1.**
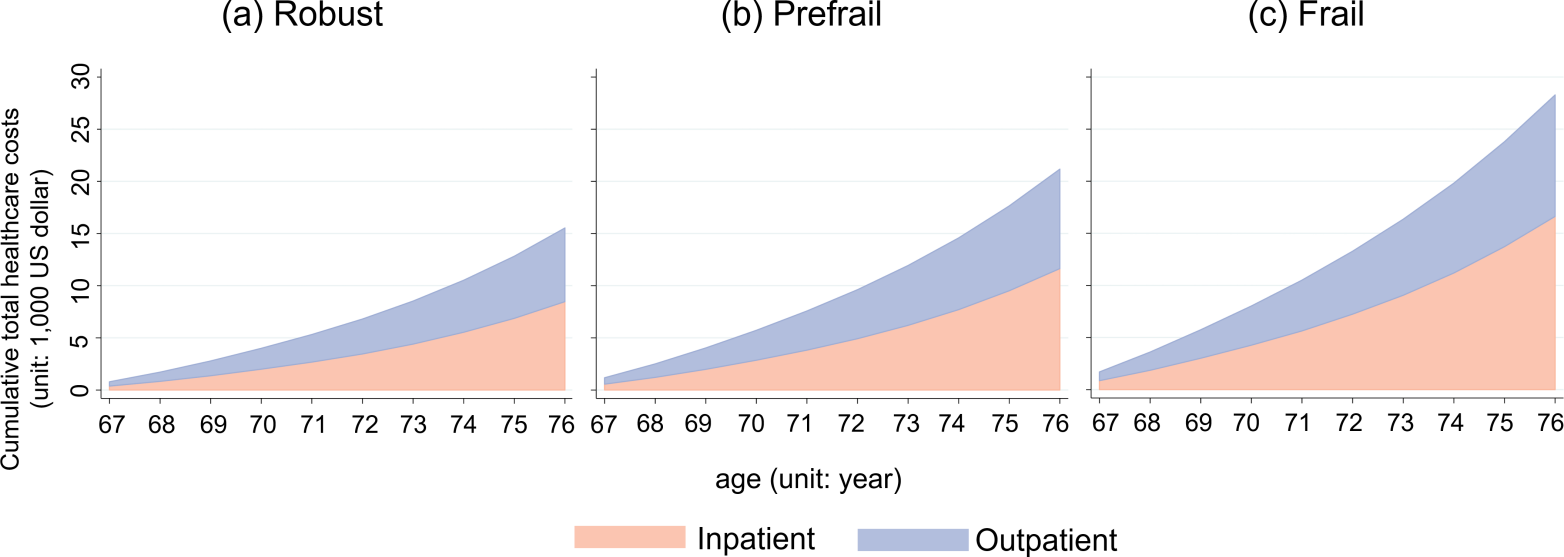
Cumulative growth in total health care costs according to frailty index at age 66 years (A) Robust; (B) Prefrail; (C) Frail categories. The X-axis represents age (years) and the Y-axis represents cumulative total health care costs (US $). The pink area represents inpatient costs and blue area represents outpatient costs.

Figure 1 shows the cumulative health care costs over 10 years by frailty category in individuals aged 66. Individuals in the frail group incurred the highest cumulative total health care costs (US $28,281), followed by the prefrail (US $23,793), and robust groups (US $19,815; *P*<.001). Compared to the robust group, the frail group was associated with higher cumulative inpatient costs (US $11,189 vs US $16,627) and outpatient costs (US $8,625 vs US $11,654) for the subsequent 10 years.

**Table 1.** Characteristics of Korean adults who participated in the National Screening Program for Transitional Ages at 66 years of age.

Characteristics	Total (N=215,887), n (%)	Frailty category^a^, n (%)			*P* value^b^
		Robust (n=123,795)	Prefrail (n=71,082)	Frail (n=21,010)	
Gender					<.001
Men	100,774 (46.68)	68,390 (55.24)	25,780 (36.27)	6604 (31.43)	
Women	115,113 (53.32)	55,405 (44.76)	45,302 (63.73)	14,406 (68.57)	
Annual income (US $)					<.001
Quartile 1 (lowest)	46,306 (21.45)	26,944 (21.77)	14,525 (20.43)	4837 (23.02)	
Quartile 2	33,002 (15.29)	19,065 (15.40)	10,716 (15.08)	3221 (15.33)	
Quartile 3	55,973 (25.93)	32,026 (25.87)	18,624 (26.20)	5323 (25.34)	
Quartile 4 (highest)	80,606 (37.34)	45,760 (36.96)	27,217 (38.29)	7629 (36.31)	
Insurance status					<.001
Employee insurance	67,302 (31.17)	37,738 (30.48)	22,880 (32.19)	6684 (31.81)	
Self-employed insurance	141,955 (65.75)	83,769 (67.67)	45,492 (64.00)	12,694 (60.42)	
Medical aid for low income	6630 (3.07)	2288 (1.85)	2710 (3.81)	1632 (7.77)	
Residential area					<.001
Capital area	77,728 (36)	44,030 (35.57)	25,886 (36.42)	7812 (37.18)	
Metropolitan area	53,811 (24.93)	30,909 (24.97)	17,487 (24.60)	5415 (25.77)	
Rural area	84,348 (39.07)	48,856 (39.47)	27,709 (38.98)	7783 (37.04)	
Examination year					<.001
2007	53,907 (24.97)	29,875 (24.13)	18,297 (25.74)	5735 (27.30)	
2008	86,427 (40.03)	48,638 (39.29)	29,050 (40.87)	8739 (41.59)	
2009	75,553 (35.00)	45,282 (36.58)	23,735 (33.39)	6536 (31.11)	

a Frailty categories were defined as robust (frailty index <0.15, prefrail (0.15 to <0.25), and frail (≥0.25).

b P values were calculated using the *χ*2 test for categorical variables.

**Figure 2.**
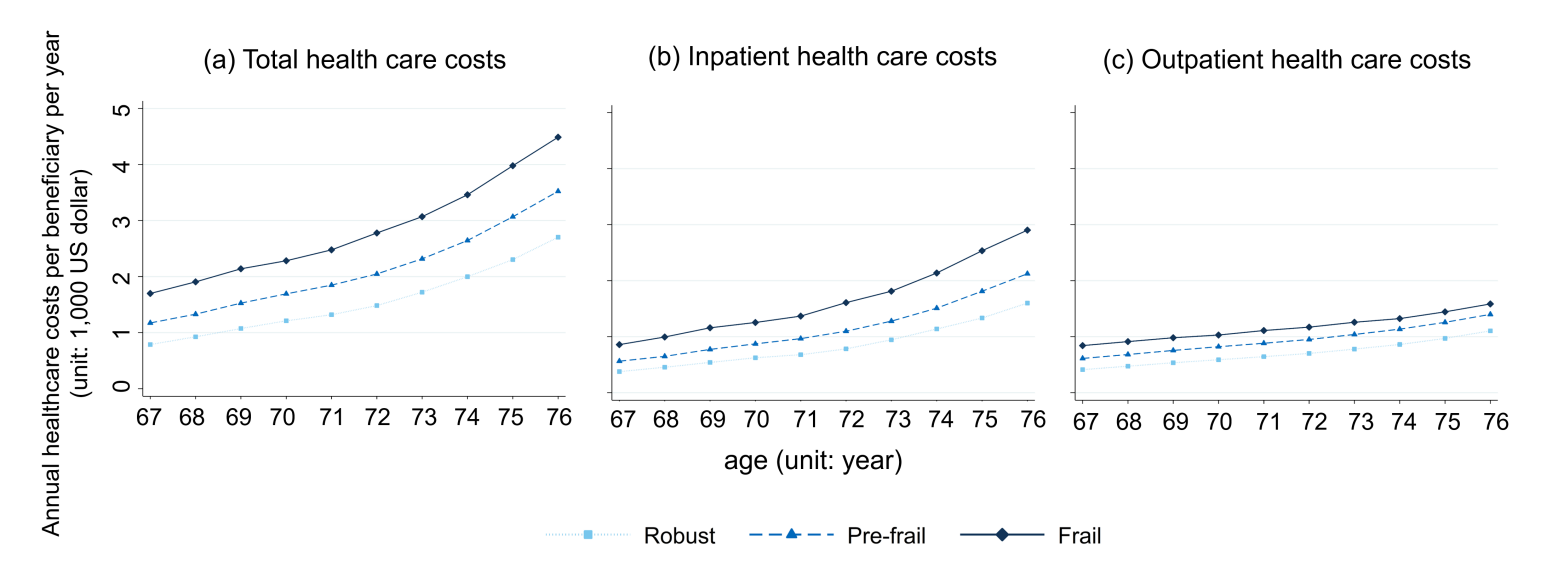
Trends in annual health care costs per beneficiary per year according to frailty index at age 66 years. The X-axis represents age (years) and the Y-axis is annual health care costs per beneficiary per year ( US dollar). The navy diamond line represents frail individuals, the blue triangle line represents pre-frail individuals, and the sky-blue square line represents robust individuals.

Over a period of 10 years, the annual growth in total health care costs, inpatient costs, and outpatient costs was greater in the frail group than in the prefrail and robust groups (Figure 2). Multivariable analyses (Table 2; Table S3 in Multimedia Appendix 1) showed that the frail group had higher mean total health care costs (difference: $827.2, SE: $20.0; *P*<.001), inpatient costs ($432.2, SE: $16.3; *P*<.001), and outpatient costs ($395.0, SE: $7.9; *P*<.001) than the robust group at baseline. In the robust group, a one-year increase in age was associated with increased total health care costs (mean change per beneficiary per year: $206.2; SE: $1.2; *P*<.001), inpatient costs ($126.8; SE: $1.0; *P*<.001), and outpatient costs ($74.4; SE: $0.4; *P*<.001). In the frail group, there were greater increases in total health care costs each year compared to the robust group (difference in the mean cost per beneficiary per year: $120.9; SE: $5.3; *P*<.001), inpatient costs ($102.8; SE: $5.2; *P*<.001), and outpatient costs ($15.6; SE: $1.5; *P*<.001). The prefrail group had higher mean total health care costs, inpatient costs, and outpatient costs than the robust group at baseline, with the annual change per one-year increase in age falling between the robust and frail groups (Table 2).

**Table 2.** Association between frailty at 66 years of age and annual health care costs and health care utilization over 10 years.^a^ results of the generalized estimating equations models that examined the association between frailty at 66 years of age and annual health care costs and health care utilization over 10 years.

Characteristics	Total health care costs		Inpatient costs		Outpatient costs		Number of inpatient days		Number of outpatient visits	
	β (SE)^b^	*P* value^c^	β (SE)	*P* value	β (SE)	*P* value	β (SE)	*P* value	β (SE)	*P* value
Frailty category^d^										
Robust	Ref.^e^		Ref.		Ref.		Ref.		Ref.	
Pre-frail	354 (8.4)	<.001	169.4 (6.4)	<.001	180.6 (3.5)	<.001	1.7 (0)	<.001	16.7 (0)	<.001
Frail	827.2 (20)	<.001	432.2 (16.3)	<.001	395 (7.9)	<.001	5 (0)	<.001	36.0 (0.1)	<.001
Year (Ref: robust)										
Per 1-year increase	206.2 (1.2)	<.001	126.8 (1)	<.001	74.4 (0.4)	<.001	0.9 (0)	<.001	2.1 (0)	<.001
Year * Frailty category										
Year * Prefrail	52 (2.4)	<.001	38.7 (2.1)	<.001	13.1 (0.7)	<.001	0.3 (0)	<.001	0.1 (0)	<.001
Year * Frail	120.9 (5.3)	<.001	102.8 (5.2)	<.001	15.6 (1.5)	<.001	0.9 (0)	<.001	-0.2 (0)	<.001

a Generalized estimation equation models were used for the analysis. The models were adjusted for the examination year, sex, annual income, insurance status, and residential area.

b β (SE) represents the regression co-efficient (β) and its standard error (SE).

c P values indicate the significance level of the comparisons.

d Frailty categories were defined as robust (frailty index<0.15), pre-frail (0.15 to <0.25), and frail (≥0.25).

e Ref. denotes the reference category used for comparisons.

The examination of health care utilization over 10 years showed that the frail group had a greater increase in inpatient days and the outpatient visits over 10 years than the prefrail or robust groups (Figure S2 and Figure S3 in Multimedia Appendix 1). The frail group had a greater mean number of inpatient days (5 d; SE: 0 d; *P*<.001) and outpatient visits (36 visits; SE: 0.1; *P*<.001) than the robust group at baseline (Table 2). In the robust group, a one-year increase in age was associated with increases in inpatient days (mean change per beneficiary per year: 0.9 d; SE: 0 d; *P*<.001) and outpatient visits (mean change per beneficiary per year: 2.1 visits; SE: 0 visits; *P*<.001). In the frail group, the number of inpatient days increased more each year compared to the robust group (difference in the mean inpatient days per beneficiary per year: 0.9 d; SE: 0 d; *P*<.001), whereas the number of outpatient visits increased to a lesser degree (difference in the mean outpatient visits per beneficiary per year: −0.2 visits; SE: 0 visits; *P*<.001). The prefrail group had a greater mean number of inpatient days and outpatient visits than the robust group at baseline, with more increases in both inpatient days and outpatient visits annually (Table 2).

In subgroup analyses, men with frailty had a greater increase in total health care costs than women with frailty (*P*-for-interaction:<.001) (Table S4 in Multimedia Appendix 1). Frail people with low-income medical aid were more likely to incur higher total health care costs than those with other insurance types and frailty groups; however, the observed difference was not significant (*P*-for-interaction: .74) (Table S5 in Multimedia Appendix 1).

## Discussion

### Principal Findings

Using a nationwide Korean cohort, we found that the frailty level at 66 years of age was associated with higher health care costs and health care utilization over the subsequent 10 years. The growth in annual health care costs and health care utilization, particularly inpatient days, was greater in individuals with frailty than in prefrail or robust individuals. Furthermore, prefrail individuals, who made up 32.9% of the population, had higher health care costs and utilization than robust individuals. Given the high prevalence of prefrailty and associated long-term health care costs and utilization, our findings suggest the importance of identifying frailty and prefrailty to control health care costs and utilization in aging populations.

Our findings are consistent with previous studies demonstrating that frailty is associated with increased use of health care resources [24-26]. A population-based cohort study showed that the association between frailty onset and increasing self-reported health care costs was prominent in inpatient care and informal nursing care [6]. A meta-analysis of 7 cohorts of community-dwelling older adults found that health care costs of prefrail and frail older adults were higher than robust individuals [27]. In addition, those who were frail faced a greater risk of hospitalization, skilled nursing facility stays [28,29], emergency department visits [30], and institutionalization [31], compared to robust individuals. Our study contributes to the existing literature by examining 10-year trajectories of health care costs and utilization in a nationally representative cohort of Koreans aged 66 years. The choice of this time point in the beginning of elderhood removes the effect of chronologic age and emphasizes the importance of early identification, prevention, and management of frailty and prefrailty. Early identification can provide the opportunity to proactively address the needs of these individuals to lower health care costs and utilization in the future [32,33]. Frailty may be prevented or delayed by interventions such as physical activity, nutrition, and comprehensive geriatric assessment [34]. Therefore, by linking frailty with long-term health care costs and utilization, our findings expand the understanding of frailty’s economic impact beyond short-term or disease-specific analyses commonly found in the literature.

Previous research suggested that incorporating frailty into a diagnosis-based model, such as the hierarchical condition category method (which is used to predict Medicare health care costs), may improve the accuracy of cost projections [35]. However, most prediction models for health care costs used in Korea do not account for frailty [36]. Although health care cost prediction models have been developed using the National Screening Program for Transitional Ages database, they have been designed for the entire population rather than specifically for older adults [37]. Our findings suggest that frailty metrics could enhance existing health care cost prediction models, providing a more nuanced understanding of health care needs in aging populations.

In our study, the associations between frailty and health care costs and utilization were stronger in men than women. Li et al [38] and colleagues found that frailty or worsening frailty had a stronger association with increased hospitalization and outpatient costs in men than in women. However, other studies did not find an interaction between frailty and sex on catastrophic health expenditures [39] or health care utilization (outpatient visit, inpatient visit, and inpatient length of stay) [40]. There are several explanations for higher health care costs among frail men than among frail women in our study. Men are generally more likely to develop serious health conditions that can be costly to manage, such as coronary heart disease, cancer, cerebrovascular disease, emphysema, cirrhosis of the liver, kidney disease, and atherosclerosis [41]. In addition, men are more likely to engage in risky health behaviors such as smoking and drinking and may be less likely to seek health care services for health issues, leading to delayed treatment [42]. These findings suggest that taking into account the differences in health risks and needs between frail men and frail women may help to improve health outcomes and reduce costs in older adults.

### Limitations

Our study had important limitations. First, we were unable to assess costs and utilization of noncovered services including the costs of outpatient prescription medications. Second, selection bias is possible due to nonparticipation of otherwise eligible individuals in the National Screening Program for Transitional Ages and deaths, which may affect the longitudinal cost trajectories. However, we previously reported no major differences in characteristics between nonparticipants and participants in the program [15]. Third, the association between frailty and health care costs and utilization may be subject to the choice of frailty definitions. Both the deficit accumulation FI and frailty phenotype have been associated with increased health care costs in previous studies [8,43]. Fourth, causality may not be inferred from our observational data. Finally, our findings may not be generalizable to other countries with different health care systems and financing structures.

### Conclusion

Our study demonstrates the potential utility of assessing frailty at 66 years of age to identify older adults who are more likely to incur high health care costs and utilize health care services in the subsequent 10 years. The long-term high health care costs and utilization associated with frailty and prefrailty call for public health approaches to prevent and manage frailty in aging populations.

## Supplementary material

10.2196/50026 Multimedia Appendix 1 Analysis of Frailty index, healthcare utilization trends, and cost variations over time.

## Abbreviations

KRW: Korean Won
