# Supplementary material for: Association of Frailty Index at 66 Years of Age with Health Care Costs and Utilization Over 10 Years in Korea: Retrospective Cohort Study
Source: JMIR Public Health Surveill. 2025 Jan 27;11:e50026. doi: 10.2196/50026 (PMC11870028; doi:10.2196/50026)
Supplement: Multimedia Appendix 1 [file publichealth-v11-e50026-s001.docx]

**MULTIMEDIA APPENDIX**

**Association of Frailty Index at 66 Years of Age with Healthcare Costs and Utilization Over 10 Years in Korea: Retrospective Cohort Study**

Jieun Jang, PhD, Anna Kim, PhD, Minji Choi, PhD, Ellen Mc Carthy, PhD, MPH, Brianne Olivieri-Mui, PhD, MPH, Chan Mi Park, MD, MPH, Jae-Hyun Kim, PhD, Jaeyong Shin, MD, MPH, PhD, Dae Hyun Kim, MD, MPH, ScD

**Table of Contents**

| **Figure S1.** | Flow Diagram of Study Population |
| --- | --- |
| **Table S1.** | Components of Deficit-Accumulation Frailty Index |
| **Table S2.** | Number of individuals at risk at each age from 67 to 76 years based on the frailty category over 10 years |
| **Table S3.** | Mean estimates of differences in annual costs between the frailty groups over time using the generalized estimating equation model |
| **Figure S2.** | Trends in annual inpatient length of stay per beneficiary per year according to frailty index at age 66 years |
| **Figure S3.** | Trends in the annual number of outpatient visits per beneficiary per year according to frailty index at age 66 years |
| **Table S4.** | Results of subgroup analysis by sex |
| **Table S5.** | Results of subgroup analysis by insurance type |

**Figure S1.** Flow diagram of the study population.

n=215,887 Enrollees who received examination between 2007 and 2009 were included in the analysis

n=435,572 Enrollees eligible for the National Screening Program for Transitional Ages at Age 66 years in 2007-2017

n=6,603 Enrollees with missing sociodemographic information

n=428,969 Enrollees with complete sociodemographic information

n=206,489 Enrollees who did not participate in the screening examination

n=1,460 Enrollees with duplicate examination records

n=5,119 Enrollees with insufficient data for frailty index calculation

n=222,480 Enrollees who participated in the screening examination

n=221,020 Enrollees with a single examination record

n=215,901 Enrollees with sufficient data for frailty index calculation were included in the analysis

n=14 Enrollees who died in the month of examination or in the month following the examination.

**Table S1.** Components of deficit-accumulation frailty index.

| **Components** | **Definition** | **Data source and measurement** | **Scoring** |
| --- | --- | --- | --- |
| **Medical history in the past year** | | | |
| Arthritis | ‘M05','M06','M08','M15','M16','M17','M19','M47','M48','M45' | Claims Data | No (0) |
|  |  |  | Yes (1) |
| Asthma | ‘J45' | Claims Data | No (0) |
|  |  |  | Yes (1) |
| Cancer | ‘C' except 'C44’ | Claims Data | No (0) |
|  |  |  | Yes (1) |
| Chronic kidney disease | ‘A1811', 'A5275', 'B520', 'C641', 'C642', 'C649', 'C689', 'D3000', 'D3001', 'D3002', 'D4100', 'D4101', 'D4102', 'D4110', 'D4111', 'D4112', 'D4120', 'D4121', 'D4122', 'D593', 'E0821', 'E0822', 'E0829', 'E0865', 'E0921', 'E0922', 'E0929', 'E102', 'E112', 'E132', 'E748', 'I120', 'I129', 'I130', 'I1310', 'I1311', 'I132', 'I701', 'I722', 'K767', 'M1030', 'M10311', 'M10312', 'M10319', 'M10321', 'M10322', 'M10329', 'M10331', 'M10332', 'M10339', 'M10341', 'M10342', 'M10349', 'M10351', 'M10352', 'M10359', 'M10361', 'M10362', 'M10369', 'M10371', 'M10372', 'M10379', 'M1038', 'M1039', 'M3214', 'M3215', 'M3504', 'N000', 'N001', 'N02', 'N03', 'N04', 'N05', 'N06', 'N07', 'N08', 'N131', 'N132', 'N1330', 'N1339', 'N14', 'N150', 'N158', 'N159', 'N16', 'N170', 'N171', 'N172', 'N178', 'N179', 'N18', 'N19', 'N250', 'N251', 'N2581', 'N2589', 'N259', 'N261', ‘'N269', 'Q6102', 'Q6111', 'Q6119', 'Q612', 'Q613', 'Q614', 'Q615', 'Q618', 'Q620', 'Q622', 'Q6210', 'Q6211', 'Q6212', 'Q6231', ' Q6232', 'Q6239', 'R944' | Claims Data | No (0) |
|  |  |  | Yes (1) |
| Congestive heart failure | ‘I50','I110','I130','I132' | Claims Data | No (0) |
|  |  |  | Yes (1) |
| Chronic obstructive pulmonary disease | ‘J40', 'J410', 'J411', 'J418', 'J42', 'J430', 'J431', 'J432', 'J438', 'J439', 'J440', 'J441', 'J449', 'J470', 'J471', 'J479' | Claims Data | No (0) |
|  |  |  | Yes (1) |
| Coronary artery disease | ‘I20', 'I21', 'I22', 'I23', 'I24', 'I25' | Claims Data | No (0) |
|  |  |  | Yes (1) |
| Diabetes | ‘E10', 'E11', 'E12', 'E13', 'E14' | Claims Data | No (0) |
|  |  |  | Yes (1) |
| Dysuria | Do you have dysuria? | Self-report | No (0) |
|  |  |  | Yes (1) |
| Fall | Have you fallen in the past 6 months? | Self-report | No (0) |
|  |  |  | Yes (1) |
| Gait disorder | Clinical observation of gait disorder | Clinical observation | No (0) |
|  |  |  | Yes (1) |
| Hearing impairment | Using pure-tone audiometry or whispered voice test (1) Pure-tone audiometry: <40db is classified as hearing loss. (2) Whispered voice test: whispers six numbers and then asks the participants to repeat. If they repeat less than three, then classified as hearing loss. | Objective measurement | No trouble (0) |
|  |  |  | Hearing loss on one side (0.5) |
|  |  |  | Hearing loss on both sides (1) |
| Hypertension | ‘H35031', 'H35032', 'H35033', 'H35039', 'I10', 'I110', 'I119', 'I12', 'I130', 'I1310', 'I1311', 'I132', 'I15', 'I674', 'N262' | Claims Data | No (0) |
|  |  |  | Yes (1) |
| Stroke | ‘G45', 'G46', 'I60', 'I61', 'I62', 'I63', 'I64', 'I65', 'I66', 'I67', 'I68', 'I69' | Claims Data | No (0 |
|  |  |  | Yes (1) |
| Vision impairment | Visual acuity testing | Objective measurement | No trouble (0) |
|  |  |  | Blindness in one eye (0.5) |
|  |  |  | Blindness in both eyes (1) |
| **Biometric or laboratory measures** | | | |
| Alanine aminotransferase, IU/L | | Laboratory test | ≤35 (0) |
|  |  |  | 36-45 (0.5) |
|  |  |  | ≥46 (1) |
| Body mass index, kg/m^2^, or weight loss | | Objective measurement | 18.5 to 24.9 (0) |
|  |  |  | 25.0 to 29.9 (0.5) |
|  |  |  | ≥30 or ≤18.5 or weight loss 5% or more per year (1) |
| Bone mineral density (T-score)* | | Diagnostic test | ≥ -1.0 (0) |
|  |  |  | -2.4 to -1.1 (0.5) |
|  |  |  | ≤ -2.5 (1) |
| Estimated GFR, ml/min/1.73m^2^ | | Laboratory test | ≥ 60 (0) |
|  |  |  | 30 to 59 (0.5) |
|  |  |  | ≤30 (1) |
| Fasting blood glucose, mg/dL | | Laboratory test | ≤ 99 (0) |
|  |  |  | 100 to 125 (0.5) |
|  |  |  | ≥ 126 (1) |
| Hemoglobin, g/dL | | Laboratory test | Men: |
|  |  |  | ≥ 13.0 (0) |
|  |  |  | 12.0 to 12.9 (0.5) |
|  |  |  | ≤ 11.9 (1) |
|  |  |  | Women: |
|  |  |  | ≥ 12.0 (0) |
|  |  |  | 11.0 to 11.9 (0.5) |
|  |  |  | ≤ 10.9 (1) |
| Systolic blood pressure, mmHg | | Objective measurement | ≤ 119 (0) |
|  |  |  | 120 to 139 (0.5) |
|  |  |  | ≥ 140 (1) |
| Total cholesterol, mg/dL | | Laboratory test | ≤ 199 (0) |
|  |  |  | 200-239 (0.5) |
|  |  |  | ≥ 240 (1) |
| **Physical health** | | | |
| Physical activity, MET-minutes/week | | Self-report | ≥ 1,017 (0) |
|  |  |  | 558 to 1,016 (0.3) |
|  |  |  | 1 to 557 (0.6) |
|  |  |  | 0 (1) |
| 3-m timed-up-and-go test, seconds | | Objective test | ≤ 10 (0) |
|  |  |  | 11 to 19 (0.5) |
|  |  |  | ≥ 20 (1) |
| **Psychological health** | | | |
| Have you dropped many of your activity or interests? | | Self-report | No (0) |
|  |  |  | Yes (1) |
| Do you feel worthless the way you are now? | | Self-report | No (0) |
|  |  |  | Yes (1) |
| Do you feel that your situation is hopeless? | | Self-report | No (0) |
|  |  |  | Yes (1) |
| Do you think your memory is inferior to your friends or colleagues? | | Self-report | No trouble (0) |
|  |  |  | Some (0.5) |
|  |  |  | A lot (1) |
| Do you think your memory has worsened over the last year? | | Self-report | No trouble (0) |
|  |  |  | Some (0.5) |
|  |  |  | A lot (1) |
| Are there times when you feel memory is an impediment when doing important things? | | Self-report | No trouble (0) |
|  |  |  | Some (0.5) |
|  |  |  | A lot (1) |
| Do others know that your memory has worsened? | | Self-report | No trouble (0) |
|  |  |  | Some (0.5) |
|  |  |  | A lot (1) |
| Do you think that, when performing daily activities, you have become clumsier than before? | | Self-report | No trouble (0) |
|  |  |  | Some (0.5) |
|  |  |  | A lot (1) |
| **Disability** | | | |
| Do you take a bath or shower without assistance? | | Self-report | No (1) |
|  |  |  | Yes (0) |
| Do you get dressed without assistance? | | Self-report | No (1) |
|  |  |  | Yes (0) |
| If a meal is prepared, do you eat without assistance? | | Self-report | No (1) |
|  |  |  | Yes (0) |
| Do you get to the toilet without assistance? | | Self-report | No (1) |
|  |  |  | Yes (0) |
| Do you prepare your own meals? | | Self-report | No (1) |
|  |  |  | Yes (0) |
| Do you get to places out of walking distance, such as shops, neighbors, hospitals, and government offices, without assistance? | | Self-report | No (1) |
|  |  |  | Yes (0) |

Reference: Jang J, Jung H, Shin J, Kim DH. Assessment of Frailty Index at 66 Years of Age and Association With Age-Related Diseases, Disability, and Death Over 10 Years in Korea. JAMA Netw Open. 2023;6(3):e2248995.

| **Table S2.** Number of individuals at risk at each age from 67 to 76 years based on the frailty category over 10 years. | | | | | | | | | | | |
| --- | --- | --- | --- | --- | --- | --- | --- | --- | --- | --- | --- |
| **Frailty category / age** | **67** | **68** | **69** | **70** | **71** | **72** | **73** | **74** | **75** | **76** | **Survival rate to age 76** |
| Robust | 123,787 | 123,212 | 122,385 | 121,481 | 120,483 | 119,315 | 118,107 | 116,709 | 115,213 | 113,631 | 91.8 |
| Pre-frail | 71,077 | 70,651 | 70,141 | 69,494 | 68,818 | 68,045 | 67,185 | 66,247 | 65,274 | 64,150 | 90.3 |
| Frail | 21,009 | 20,795 | 20,520 | 20,211 | 19,884 | 19,536 | 19,151 | 18,747 | 18,350 | 17,903 | 85.2 |
| **Total** | 215,873 | 214,658 | 213,046 | 211,186 | 209,185 | 206,896 | 204,443 | 201,703 | 198,837 | 195,684 | 90.6 |

*Frailty categories were defined as robust (frailty index <0.15), pre-frail (0.15 to <0.25), and frail (≥0.25) from the screening examination at age 66 years.

**Table S3.** Mean estimates of differences in annual costs between the frailty groups over time using the generalized estimating equation model.

|  | Total healthcare costs | | | | | Inpatient costs | | | | | Outpatient costs | | | | |
| --- | --- | --- | --- | --- | --- | --- | --- | --- | --- | --- | --- | --- | --- | --- | --- |
| Age | Robust (a) | Pre-frail (b) | Frail (c) | Difference between robust and pre-frail (b)-(a) | Difference between robust and frail (c)-(a) | Robust (a) | Pre-frail (b) | Frail (c) | Difference between robust and pre-frail (b)-(a) | Difference between robust and frail (c)-(a) | Robust (a) | Pre-frail (b) | Frail (c) | Difference between robust and pre-frail (b)-(a) | Difference between robust and frail (c)-(a) |
| 67 | 739 | 1,084 | 1,571 | 345 | 833 | 351 | 503 | 771 | 151 | 419 | 396 | 589 | 814 | 194 | 418 |
| 68 | 945 | 1,342 | 1,898 | 397 | 953 | 478 | 668 | 1,000 | 190 | 522 | 470 | 677 | 904 | 207 | 434 |
| 69 | 1,151 | 1,599 | 2,224 | 449 | 1,073 | 604 | 833 | 1,229 | 229 | 624 | 545 | 765 | 994 | 220 | 450 |
| 70 | 1,357 | 1,857 | 2,551 | 501 | 1,194 | 731 | 998 | 1,457 | 267 | 726 | 619 | 852 | 1,084 | 233 | 465 |
| 71 | 1,563 | 2,115 | 2,877 | 552 | 1,314 | 857 | 1,163 | 1,686 | 306 | 829 | 694 | 940 | 1,174 | 246 | 481 |
| 72 | 1,768 | 2,373 | 3,203 | 604 | 1,434 | 984 | 1,328 | 1,915 | 344 | 931 | 768 | 1,028 | 1,264 | 259 | 496 |
| 73 | 1,974 | 2,630 | 3,529 | 656 | 1,555 | 1,110 | 1,493 | 2,143 | 383 | 1,033 | 843 | 1,115 | 1,354 | 273 | 512 |
| 74 | 2,180 | 2,888 | 3,855 | 708 | 1,675 | 1,237 | 1,658 | 2,372 | 421 | 1,135 | 917 | 1,203 | 1,445 | 286 | 527 |
| 75 | 2,386 | 3,145 | 4,181 | 759 | 1,795 | 1,363 | 1,823 | 2,601 | 460 | 1,238 | 992 | 1,291 | 1,535 | 299 | 543 |
| 76 | 2,592 | 3,403 | 4,507 | 811 | 1,916 | 1,489 | 1,988 | 2,829 | 498 | 1,340 | 1,066 | 1,378 | 1,625 | 312 | 558 |

Frailty categories were defined as robust (frailty index <0.15), pre-frail (0.15 to <0.25), and frail (≥0.25) from the screening examination at age 66 years. Models were adjusted for examination year, sex, annual income, insurance status, and residential area.

**Figure S2.** Trends in annual inpatient length of stay per beneficiary per year according to frailty index at age 66 years.


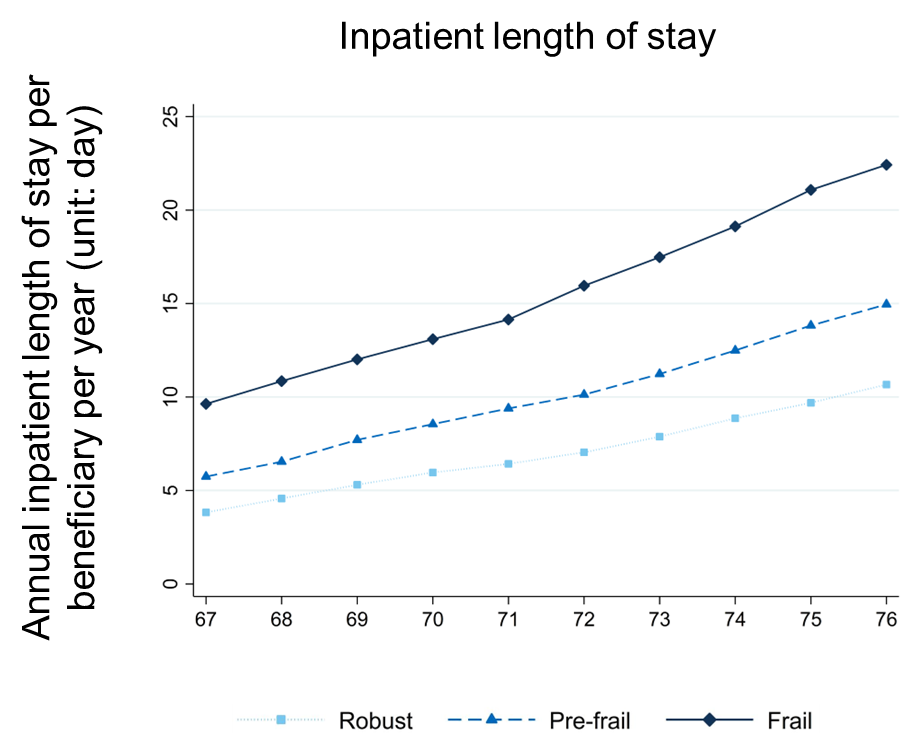


*Frailty categories were defined as robust (frailty index <0.15), pre-frail (0.15 to <0.25), and frail (≥0.25) from the screening examination at age 66 years. The x-axis is age (unit: year) and the y-axis is inpatient length of stay (days). The navy diamond line represents frail older adults, the blue triangle line represents pre-frail older adults, and the sky-blue square line represents robust older adults.

**Figure S3.** Trends in the annual number of outpatient visits per beneficiary per year according to frailty index at age 66 years.

**
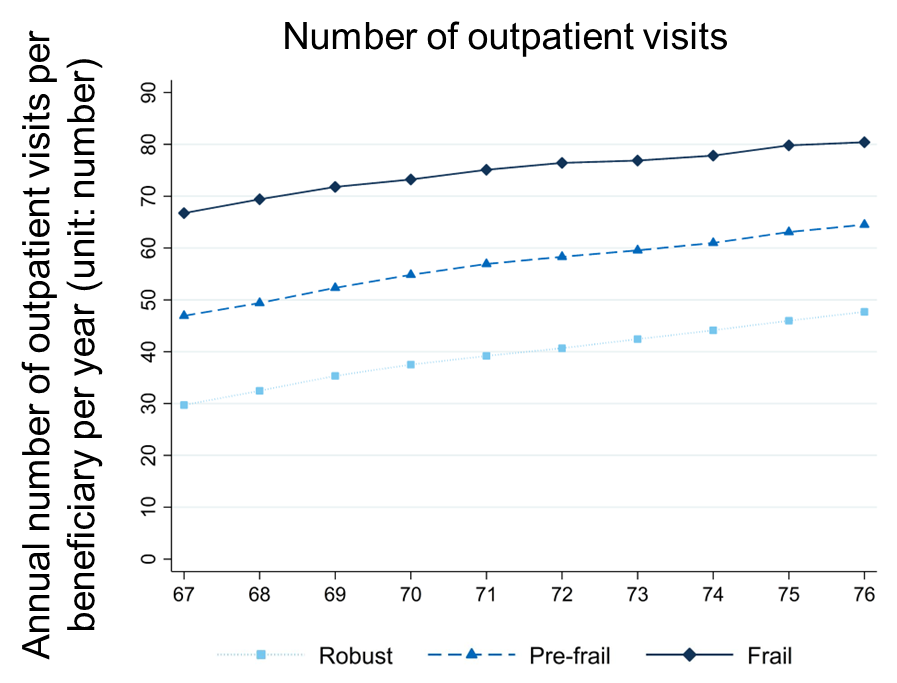
**

*Frailty categories were defined as robust (frailty index <0.15), pre-frail (0.15 to <0.25), and frail (≥0.25) from the screening examination at age 66 years. The x-axis is age (unit: year) and number of outpatient visits (n). The navy diamond line represents frail older adults, the blue triangle line represents pre-frail older adults, and the sky-blue square line represents robust older adults.

**Table S4.** Results of subgroup analysis by sex.

| **Characteristics** | | **Men** | | | **Women** | | |
| --- | --- | --- | --- | --- | --- | --- | --- |
|  |  | ***β*** | **S.E.** | ***P*-value** | ***β*** | **S.E.** | ***P*-value** |
| **Frailty category** | |  |  |  |  |  |  |
|  | Robust | Ref. |  |  | Ref. |  |  |
|  | Pre-frail | 411.6 | 15.2 | <0.001 | 312.1 | 9.9 | <0.001 |
|  | Frail | 913.2 | 40.6 | <0.001 | 774.7 | 22.2 | <0.001 |
| **Time (Robust=Ref.)** | | 226.9 | 1.8 | <0.001 | 183.7 | 1.5 | <0.001 |
| **Time x Frailty category** | |  |  |  |  |  |  |
|  | Time * Pre-frail | 81.3 | 4.6 | <0.001 | 49.7 | 2.7 | <0.001 |
|  | Time * Frail | 171.3 | 11.8 | <0.001 | 116.6 | 5.7 | <0.001 |

^1^Frailty categories were defined as robust (frailty index <0.15), pre-frail (0.15 to <0.25), and frail (≥0.25) from the screening examination at age 66 years. The results were adjusted for examination year, sex, annual income, insurance status, and residential area.

^2^The observed difference was significant (*P*-value for homogeneity: <0.001).

**Table S5.** Results of subgroup analysis by insurance type.

| **Characteristics** | | **Employee insurance** | | | **Self-employed insurance** | | | **Medical aid for low income** | | |
| --- | --- | --- | --- | --- | --- | --- | --- | --- | --- | --- |
|  |  | ***β*** | **S.E.** | ***P*-value** | ***β*** | **S.E.** | ***P*-value** | ***β*** | **S.E.** | ***P*-value** |
| **Frailty category** | |  |  |  |  |  |  |  |  |  |
|  | Robust | Ref. |  |  | Ref. |  |  | Ref. |  |  |
|  | Pre-frail | 341.0 | 14.6 | <0.001 | 359.1 | 10.5 | <0.001 | 499.7 | 53.4 | <0.001 |
|  | Frail | 836.3 | 34.9 | <0.001 | 804.8 | 25.0 | <0.001 | 1239.7 | 85.8 | <0.001 |
| **Time (Robust=Ref.)** | | 207.4 | 2.1 | <0.001 | 203.7 | 1.4 | <0.001 | 330.0 | 10.3 | <0.001 |
| **Time * Frailty category** | |  |  |  |  |  |  |  |  |  |
|  | Time * Pre-frail | 45.1 | 4.1 | <0.001 | 52.0 | 2.9 | <0.001 | 65.6 | 15.9 | <0.001 |
|  | Time * Frail | 116.0 | 9.3 | <0.001 | 114.2 | 6.7 | <0.001 | 132.8 | 23.0 | <0.001 |

^1^Frailty categories were defined as robust (frailty index <0.15), pre-frail (0.15 to <0.25), and frail (≥0.25) from the screening examination at age 66 years. The results were adjusted for examination year, sex, annual income, insurance status, and residential area.

^2^The observed difference was not significant (*P*-value for homogeneity: 0.739).
